# Moving Past the One-Size-Fits-All Education-Training Model of Police Academies to the Self-Prescribed Individualized Exercise Prescription Model

**DOI:** 10.3390/ijerph182111676

**Published:** 2021-11-07

**Authors:** Zacharias Papadakis, Andreas Stamatis, Filip Kukic, Nenad Koropanovski

**Affiliations:** 1 Human Performance Laboratory, Barry University, Miami Shores, FL 33161, USA; 2 SUNY Plattsburgh, Plattsburgh, NY 12901, USA; astam004@plattsburgh.edu; 3 Police Sports Education Center, Abu Dhabi Police, Abu Dhabi 253, United Arab Emirates; filip.kukic@gmail.com; 4 Department of Criminalistics, University of Criminal Investigation and Police Studies, 11080 Belgrade, Serbia; nenad.koropanovski@kpu.edu.rs

**Keywords:** education-training, ability-based training, conditioning, tactical

## Abstract

Law enforcement agencies generally employ the “one-size-fits-all” education-training model. Its effectiveness compared to alternative training models has been under scrutiny. Physical fitness scores of Serbian male (n = 98) and female (n = 79) police cadets during their yearly evaluation were compared. Cadets trained for the first 3 years with the “one-size-fits-all” model. In the fourth year, they self-prescribed an individualized exercise program based on the obtained curriculum knowledge. A two-way MANOVA revealed a significant effect of academic years on combined variables (*p* < 0.001) and significant differences between academic years for deadlift, half squat, standing long jump, sit-ups and 12-min Cooper test time (*p* < 0.001). Sex also had a significant main effect on combined variables (*p* < 0.001) with males outscoring females on all of the fitness assessments. For pull-ups, there was a significant year * sex interaction (*p* = 0.01) with the third year to be pivotal for female and male performance, respectively. In conclusion, the use of a “one-size-fits-all” model, presented differences in physical fitness scores between the years one to three, pointing to its questionable effectiveness. On the contrary, the self-prescribed individualized exercise program of the fourth year elicited greater fitness scores, indicating the need to evaluate the applicability of such a training model more.

## 1. Introduction

Law enforcement officers’ (LEOs) occupational demands require certain levels of physical fitness [1,2,3]. Low levels of physical fitness may impede LEOs’ capacities to undertake operational tasks [3], such as suspects’ pursuit and apprehension [4] and use of deadly force when they discharge their firearms [5]. Police academies have the responsibility to physically prepare their graduates for the police-related occupational demands [1,6,7,8,9,10,11,12,13]. On top of that, police academies have the responsibility to build such fitness habits that will support both cadets’ health and fitness not only during their academy training but also throughout their career [1,6,7,8,9,10,11,12,13].

In general, certain police academies accept their cohorts every year based on standardized evaluations of their fitness levels [14]. Since cadets, in general, follow the same training program from the first year until their senior year, they may have the same fitness levels throughout the years of being in the police academy [15]. Lockie et al. [15] examined differences in fitness levels among three classes in the largest USA law enforcement agency (LEA). The authors reported that selected fitness test scores, such as in push-ups, sit-ups, arm ergometer revolutions, 75-yard pursuit and 2.4-km run time, did not differ between the classes. Similar results were reported when fitness data from custody assistant academy classes were analyzed [16], pointing out that the applied training programs may not be different [15]. However, even in this case, individual differences in physical characteristics within classes should be taken into consideration for optimal training adaptations and overall fitness improvements [6,15,16].

Based on the established theoretical and methodological principles of training [17] and the available literature on tactical athletes, police academies should ideally move past the “one-size-fits-all” training model [1,18,19]. One-size-fits-all training approach means that irrespective of any pre-existing fitness or ability levels, all the cadets within an academy class will have to follow the same standardized training program [16]. Such an approach though may be inappropriate for certain cadets and may jeopardize their health status, simply just because of inherent sex fitness differences and training adaptations [6,16]. LEAs typically operate under a non-discriminatory hiring policy regarding sex/gender [20]. When cadets’ occupational capability or survivability of training are assessed, uniform tests are used that do not differ according to sex/gender, with identical performance standards for both sexes/genders [8,21,22,23,24,25,26]. Sex differences in physical performance are well documented, with males outperforming females [6,12,15,24,27,28,29]; therefore, using a one-size-fits-all approach may be considered discriminatory in respect to sex/gender.

However, despite such available evidence [16], the currently applied training programs are not prescribed in an individualized way [30]. Such programs expect all recruits to respond and adapt to training stimuli in a similar way [6,18,19] with loads and intensities to reflect the overall class’ fitness levels [16]. This practice is based on the premise that all cadets will be asked to perform the same LEO duties upon their graduation; therefore, their training must be the same. Nevertheless, such practice may hinder their occupational performance, impose health issues and put their status in the academy at risk [18,19,31,32,33]. For example, it has been shown that having a periodized training program, tailored to each recruit’s needs may prevent voluntary dropouts due to recruits’ inability to cope with the imposed stress [6]. Moreover, individualized ability-based training programs have been shown to have comparable or even better fitness gains to group training (e.g., one-size-fits- all) [7,18,34,35].

It has been suggested that further analysis on fitness levels among academies and their respective profiles [7,15,16] would provide more evidence on individualized exercise prescription geared toward the improvement and maintenance of both cadets’ and LEOs’ fitness levels [12]. The vast majority of the available literature examines the fitness characteristics of cadets–officers in LEAs located in the USA. There is scarce overseas research examining cadets’ fitness characteristics and LEOs, with the majority of the published research representing specific Serbian and Abu Dhabi police academies [36,37,38,39].

No matter the origin of the information and existing differences between LEAs from different countries [40], LEAs need to develop curricula that prepare cadets’ and LEOs’ occupational workload both during their academy years and throughout their career, so their fitness levels do not decline upon graduation, allowing and ensuring their health and wellness is promoted [12,41,42]. Most of the time, LEAs have their own curricula that are dictated by their respective needs and local laws. That creates gaps and inefficiencies with strength and conditioning for police academy training [43].

In many cases, agencies implement monolithic one-size-fits-all strength and conditioning programs without adjusting to the cadets’ fitness level. In other, individual cases, LEAs implement ability-based training. Although both approaches have pros and cons, with ability-based training being more favorable, they have the same downfall, which is sustainability. Both approaches are command-based, meaning that cadets do what, how and how much they are told to do. Although this is shown to improve cadets’ fitness [44,45,46,47], it is less likely to provide a long-term retention of cadets (later officers) in regular physical activity (PA) or exercise. Studies on police cadets and officers showed a reduction in PA and fitness with the time spent in service [48,49].

Another approach to the physical preparation of cadets could include one of the above mentioned combined with education on understanding how to implement exercise on their own, when it is not provided by the academy and/or LEA. For example, in Serbia, curricula are built up to cadets’ third year. As a result, during their fourth year, cadets utilize the previously obtained knowledge by applying different training methods on their own. In more detail, curricula include three training classes mostly based on self-defense and use of force, while strength and conditioning take a small portion of curricula and includes teaching on basic methods of physical preparation [44,50]. In their junior year, cadets prescribe their own individualized exercise training program. Such practice though, may initially lead to smaller or no acute effects on physical fitness [16,39,51]. However, it would allow them to eventually organize their individualized and ability-based training models that have been suggested as alternative practices to the one-size-fits-all approach for LEAs in order to enhance fitness levels and prevent injuries [18,30,52,53,54,55,56]. In general, such practices are still not common in cadets’ education nor in LEAs. To the best of our knowledge, only one study stated that the training staff of a USA LEA allowed its recruits to develop their own program in the final weeks of the academy [9].

Moreover, presenting evidence on LEAs’ practices in countries other than the US will allow for knowledge dissemination regarding exercise prescription between LEAs and their respective training practices at a larger scale for (a) the betterment of cadets and LEOs successful training [57] by having careers with lower injury risks [13], and (b) improved psychological and physical health [42,58,59]. Minimal research with adequate sample sizes to establish normative physiological profile data within this population exists [12]. On top of that, sex/gender disparities in LEAs are a fact, but not enough research on female/women recruits/cadets exists as well [8,23,60], and further research is required in this area.

Therefore, based on the available literature [9,12,15,16,24,61], the aim of this study was to document the physical fitness profile of a specific LEA from Serbia during their academy years under the applied training curriculum. It was hypothesized that (a) the training curriculum will produce different fitness scores between the academy classes of the Serbian Police cadets (freshman, sophomore, junior and senior) with a difference observed between the one-size-fits-all training-education approach of the first, second and third academy years and the fourth year when a self-prescribed individualized exercise program is applied, and (b) the male cadets’ fitness scores will be greater compared to the female ones, irrespective of their academic year.

## 2. Materials and Methods

### 2.1. Experimental Approach to the Problem

A retrospective analysis of pre-existing physical fitness data from the University of Criminal Investigation and Police Studies (UCIPS) in Belgrade, Serbia was conducted. Data were collected by the same specialized physical education professors of UCIPS (i.e., PhD in Sports Science) during the regular specialized physical education classes at the UCIPS’s training facility, following the LEAs’ established policies and procedures for data collection. The UCIPS curriculum includes four Specialized Physical Education (SPE) classes per week, one theoretical and three practicals. The teaching program of the SPE is a one-semester course during the first three years of studies, with 180 class hours in total. The first three theoretical and five practical classes include teaching and exercising relative to fitness. During each academic year, SPE curriculum is covered in three semesters with 60 class hours (45 practical and 15 theoretical), including, predominantly, martial arts training (Karate, Judo, Jujutsu) in forms of defensive tactics and use of force. The students are taught through the following three levels: (a) becoming familiar with basic techniques (SPE 1); (b) their use in controlled conditions (SPE 2); and (c) their situational usage (SPE 3). Strength and conditioning take a small amount of the classes at the beginning of the studies (first semester), when the students are introduced to basic methods of physical preparation. Through this training, cadets need to achieve specific fitness levels, as testing on fitness capacities is a part of their examination and achieving a minimum of a defined level is mandatory.

As part of their yearly evaluation and progress on the program, cadets complete a series of physical fitness tests (i.e., a maximal force of handgrip, deadlift, half-squat, standing long jump, pushups, sit-ups, pull-ups and 12-min running test) as dictated by the UCIPS Educational Committee, up to their senior year. During the senior year, cadets utilize the curriculum-related obtained knowledge on exercise science and strength and conditioning to construct their own training programs in order to achieve the minimum defined levels to graduate from the academy.

A comparative analysis of their annual physical fitness testing per academic year and sex was used to address the aims of this study. Researchers did not control the components of the one-size-fits-all training education program or the self-prescribed individualized exercise programs. Additionally, no control was placed on other lifestyle common practices that were applied in this LEA, such as dietary interventions, sleep and work schedule.

### 2.2. Subjects

The convenience sample of different academic cohorts was comprised of 177 cadets, 98 males (age, 20.6 years ± 1.3 SD; height, 183.3 cm ± 6.5 SD; weight, 82.6 kg ± 9.2 SD) and 79 females (age, 20.9 years ± 1.4 SD; height, 170.6 cm ± 4.6 SD; weight, 63.9 kg ± 6.4 SD). The procedures were conducted with the permission of the respective Ethics Committee (# 440-2, 2019) to use pre-existing data and all study practices were conducted according to the Declaration of Helsinki.

### 2.3. Procedures

#### 2.3.1. Hand Grip

Maximal force of the left- and right-hand grip was assessed following the procedures previously described in research [62,63]. In short, cadets were in a sitting position, with the arm slightly flexed (about 160–170° between the forearm and upper-arm) and slightly abducted, just so the upper-arm does not touch the body. Cadets, using a standardized familiarization protocol, were allowed to familiarize with the test by performing several attempts. After the familiarization, they were provided with three to five minutes of rest, and then they performed the test. They were instructed to squeeze the dynamometer as strong as possible for five seconds, without connecting the arm to the body or flexing the elbow. They had three maximal trials and the strongest trial was used for the analysis. During the test, they could see a graphical display of their force development and they were verbally encouraged by the tester. The hand grip force was collected using a dynamometric probe fixed to the to the ground via chain. The sampling frequency of the probe was 500 Hz, which was transferred from analog to digital signal using the software (Isometrics, ver. 3.1.1, Belgrade, Serbia).

#### 2.3.2. Deadlift

Maximal force back and hip extensors in a dead lift were assessed using a standardized measurement procedures reported in previous research [64]. Subjects were in the position of isometric dead lift, pulling the tensiometric probe with a built-in A/D converter connected to a software system (Isometrics, ver. 3.1.1, Belgrade, Serbia). A subject stood on the platform, grasped the bar in front at shoulder width, while in slight hip flexion, with the upper body in a neutral position. Feet were in a parallel position, shoulder-width apart. After the signal was given, the participant executed maximal voluntary isometric contraction by attempting to extend the lower back with as much force as possible, with no movements made in the front and lateral planes. The participants were encouraged verbally, and they had live visual feedback of their force development.

#### 2.3.3. Half-Squat

Cadets were in the position of isometric half-squat, pulling the tensiometric probe with a built-in A/D converter connected to a software system (Isometrics, ver. 3.1.1, Belgrade, Serbia). A subject stood on the platform, grasped the bar behind the hamstrings at shoulder width, while in position of half squat, with the upper body in a neutral position. Feet were in a parallel position, shoulder-width apart. After the signal was given, the participant executed maximal voluntary isometric contraction by attempting to extend legs with as much force as possible, with no movements were made in the front and lateral planes. The participants were encouraged verbally, and they had live visual feedback of their force development.

#### 2.3.4. Standing Long Jump

Lower body power in the horizontal plane was assessed using a standing long jump test following previously described procedures [11,65]. The participant was instructed to jump as far as possible from the marked line with both feet, while the hands were free to swing. The distance from the starting to the landing point at the heel contact was measured in centimeters with 1-cm measurement precision [66].

#### 2.3.5. Push-Ups

The repetitive arm extensor power was estimated with a test of the maximum number of push-ups performed within 10 s [11]. The initial position was with the body prone, arms extended, hands positioned at shoulder width and only feet and palms touching the floor [67]. From the initial position, the participant went down with his/her chest to the ground, bending only the elbows, while the body remained in the firm, starting position. The results were expressed in a number of correctly performed push-ups.

#### 2.3.6. Sit-Ups

The abdominal flexor repetitive power was estimated as the number (#) of sit-ups in 30 s, with alternate rotations of the upper body to the left and right and contact between the opposite knee and elbow [11]. The cadets were laying on their back with their knees bent at 90°, feet fixed flat on the ground, palms crossed behind the head and the elbows wide open. The cadets performed an abdominal flexion with trunk rotation to one side, returned to the starting position and then abdominal flexion with the rotation to the other. The results were expressed in a number of correctly performed sit-ups.

#### 2.3.7. Pull-Ups

Upper body pulling strength was evaluated by performing 10 repetitions for females and maximum repetitions for males as previously described by law enforcement personnel [68]. Briefly, cadets were required to hang on a bar in a vertical position using a shoulder’s width apart pronated grip with their arms fully extended. While cadets maintained their vertical alignment, they had to pull themselves up until their chin was positioned over the bar, which was counted as one repetition. Then, they had to descend in a controlled manner with arms to be fully extended and they had to continue until they failed to raise their chin above the bar.

#### 2.3.8. Cooper Test (12 Min)

General aerobic endurance was estimated using a 12-min Cooper running test. The cadets were required to run around the 400-m-long circuit track and cover the longest possible distance in 12 min [44]. During the test, the participants were verbally encouraged and motivated in order to minimize the pacing.

## 3. Statistical Analysis

Dependent variables included the physical fitness test scores (i.e., eight fitness tests), while independent variables included the academic years (first—Freshman, second—Sophomore, third—Junior and the fourth—Senior) and the sex (male—female). All the dependent variables have been part of a physical fitness test and we wanted to be able to detect possible differences on the combination of the dependent variables due to the intercorrelation. Therefore, a two-way Multivariate Analysis of Variance (MANOVA) was deemed as a more appropriate test to be performed.

Due to the retrospective nature of the study, a power analysis was used to determine whether or not our sample was adequate. Using G * Power for Mac (vs. 3.1.94, 2009) and selecting F-test; MANOVA special effects and interactions with f2 (V) = 0.0625–medium effect; alpha levels at 0.05; power at 0.80; # of groups, eight; number of predictors, two; and response variables, eight; yielded a total minimum sample size of 162 [69]. The data were explored for outliers and multivariate normality using boxplots and histograms and proper corrections were applied in case of violations [70]. The sample size was large (*N* = 177) and the Pillai–Bartlett trace was used due to its robustness to violations of assumptions.

As the group sizes were unequal (Freshman n = 33; Sophomore n = 91; Junior n = 38; Senior n = 15), the homogeneity of covariance matrices was checked to verify multivariate normality [70]. In addition, an outliers check was performed, as in fairly large samples, outliers are a more pressing concern than normality. Three outliers were identified using Mahalanobis’s distance and were removed from the subsequent analysis with a final analyzed sample of *N* = 174 [70]. Bonferroni post hoc procedures were used to follow-up the significant findings.

Standardized effects sizes (ES) were also calculated with the following threshold values: <0.2, trivial; >0.2, small; >0.6, moderate; and >1.2, large [71]. The statistical significance for this study was set a priori with a *p* value ≤ 0.05. The group characteristics were reported as mean ± SD and data analyses were completed with the IBM Statistical Package for Social Sciences (SPSS) for Mac, v. 26, (IBM Corp., Armonk, NY, USA).

## 4. Results

The descriptive statistics for age, height and weight per academic year and sex are presented in Table 1. The descriptive statistics for all the examined variables per academic year and sex are presented in Table 2.

Using Pillai’s trace, there was a significant effect of academic years on the combined dependent variables, V = 0.49, F(24, 483) = 3.90, *p* < 0.001, η^2^ = 0.16. Separate univariate ANOVAs on the outcome variables revealed a significant effect of academic years only on the deadlift (F(3, 166) = 3.74, *p* = 0.01, η^2^ = 0.06), half-squat (F(3, 166) = 9.74, *p* < 0.001, η^2^ = 0.15), standing long jump (F(3, 166) = 7.71, *p* < 0.001, η^2^ = 0.12), sit ups (F(3, 166) = 6.38, *p* < 0.001, η^2^ = 0.10) and on the 12-min Cooper test time (F(3, 166) = 3.95, *p* = 0.01, η^2^ = 0.07). The post hoc analysis using Bonferroni correction is presented in Table 3.

Using Pillai’s trace, there was a significant effect of sex on the combined dependent variables, V = 0.92, F(8, 159) = 232.55, *p* < 0.001, η^2^ = 0.92. Separate univariate ANOVAs on the outcome variables revealed a significant effect of sex on all of the dependent variables (i.e., handgrip strength, F(1, 166) = 576.03, *p* < 0.001, η^2^ = 0.78; the deadlift, F(1, 166) = 375.69, *p* < 0.001, η^2^ = 0.69; half-squat, F(1, 166) = 401.79, *p* < 0.001, η^2^ = 0.71; standing long jump, F(1, 166) = 404.76, *p* < 0.001, η^2^ = 0.62; push-ups, F(1, 166) = 267.08, *p* < 0.001, η^2^ = 0.62; sit ups, F(1, 166) = 69.78, *p* < 0.001, η^2^ = 0.30; pull-ups, F(1, 166) = 323.86, *p* < 0.001, η^2^ = 0.66; and on the 12-min Cooper test time, F(1, 166) = 276.33, *p* < 0.001, η^2^ = 0.62). Sex related pairwise comparisons are presented in Table 4.

**Figure 1:**
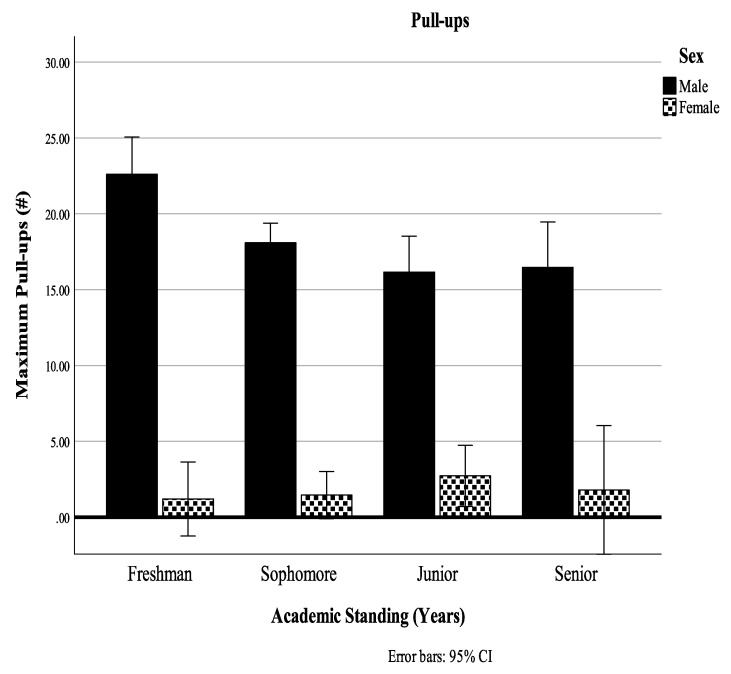
Pull-ups Academic Years by Sex Interaction. # Denotes number of performed repetitions for the respective exercises.

Using Pillai’s trace, there was a significant effect of year X sex interaction on the combined dependent variables, V = 0.22, F(24, 483) = 1.60, *p* = 0.04, η^2^ = 0.07. Separate univariate ANOVAs on the outcome variables revealed a significant effect of year X sex interaction only on pull-ups, F(3, 166) = 4.04, *p* = 0.01, η^2^ = 0.07. Male freshman presented the highest number of executed pull-ups, showing a gradual decline for the next two years and performance stabilization for their senior year. Female juniors outperformed the freshmen and sophomores, with seniors to present higher values than the freshmen and sophomores but still lower than the juniors (Figure 1).

## 5. Discussion

This study documented the physical fitness profile of specific Serbian LEA academy classes based on the applied exercise training curriculum (i.e., one-size-fits-all vs. individualized exercise prescription). The results supported our hypotheses (a) as males outperformed females at every fitness related test, and (b) fitness scores, as documented in many of the related tests, were not only different between years one to three (i.e., one-size-fits-all approach/paramilitary model of academy training), but also when compared to the last year when an individualized approach was used (i.e., customized exercise prescription based on the education that cadets obtained through the curriculum). Four distinct trends between the academic years were observed and will be discussed accordingly under the one-size-fits-all and individualized exercise prescription point of view.

For the standing long jump and 12-min Cooper test, cadets during their freshman year presented the highest performance. At their second year, their performance was the lowest and gradually, during their third year, they started to recover. This recovery process was completed during their last and fourth year when their performance was around the same as their first. This trend can be explained due to the fact that cadets were training in order to join the academy. Being fit is part of the hiring process and having the prerequisite physical abilities is a necessity to complete the academy training and the related occupational demands [6,10,29,72,73]. Therefore, first-year cadets were already fit and the applied training program (i.e., one-size-fits-all) maintained their fitness levels. However, during the second year, the decline in their respective performance may be explained by environmental stressors (e.g., relocation and unique living accommodations), physical stressors (e.g., intensive training with poor sleep and lack of proper time for recovery) [13] and mental stressors [59,74] that may have led to overtraining [75], injury [76] and illness [77] and even separating from the academy [6,73]. In addition, the drop in these fitness assessments may have been due to the specific structure of the curriculum of this specific LEA. It is possible that the weekly frequency of training sessions implemented to improve cadets’ physical fitness could be insufficient to elicit the expected job- and knowledge-related adaptations [78]. In this case, the applied training program was not a potent stimulus to counteract the aforementioned factors and failed to maintain the cadets’ previously obtained fitness levels. The gradual recovery of the fitness levels during the third year though, may be attributed to the fact that the cadets with the continuing applied training program became more accustomed to the environmental, physical and mental stressors that they were facing during the first and second years, building resilience and coping better with the stressors [60]. The fact that during the fourth year the fitness scores returned to levels similar to the first year shows that the one-size-fits-all approach and the individualized exercise prescription do not differ on the magnitude of the absolute adaptations they can elicit. Probably though, it highlights the effectiveness of the individualized exercise prescription, which was able to significantly improve the cadets’ performance in comparison to their junior year.

For the deadlift, push-ups and half-squat performance, cadets showed a gradual increase in their performance from year one to year four. In this case, both training approaches (i.e., one-size-fits-all vs. individualized exercise prescription) proved their effectiveness. The cadets’ performance kept increasing, maintaining the observed momentum from the first year as part of the elicited and anticipated physiological adaptations from year to year [17,79,80].

For the pull-ups performance though, cadets presented the opposite trend; a gradual decline in their performance from year one to year four. This is something that is definitely alarming, and it shares the reasoning of the previous trend, as the most anticipated outcome would have been a maintenance or an increase in fitness capacities as a result of the training during the academy years [17,79,80]. In addition to that, a year * sex interaction was present, where the first-year males were able to perform the maximum number of pull-ups, showing a gradual decline for the next two years and performance stabilization for their senior year. The third-year females outscored the first- and second-year ones, with the fourth-year female cadets presenting higher values than the first and second years but still lower than the third years.

For the sit-up performance, a unique trend was revealed as the cadets’ performance dropped in the second year compared to the first. During the third year, the cadets maximized their performance, which again was dropped to the first-year levels during their fourth year. The reasons behind the trend for years one to three were covered earlier, as it shares characteristics with previous patterns. However, the drop in the fourth year may be attributed to a lack of focus on core strength as part of the individualized exercise prescription program. The issue suggests that most of the time, core strengthening exercises are not explicitly stated, as in the current national guidelines, where they fall under the strength training and balance exercises, respectively [81].

Even though it is not possible for the researchers to know the cadets’ level of physical fitness before joining the academy, it is logical to assume that since they entered the academy, they met the minimal requirements for entry and they probably trained using the same methods due to the standardized entry testing [7,10,15]. Therefore, recognizing that between this homogenous group we may have some relatively “untrained vs. trained and/or high-responders- vs. low-responders” to standardized training, may explain the different performance trends in physical fitness scores that we observed [82]. This phenomenon is also highlighted by the large variation in the mean responses that were observed in many of the examined variables, pointing out that the one-size-fits-all model of training was not that optimal [51,52]. In addition, the great spread in the documented responses indicate that the training program elicited adaptations to both aerobic and anaerobic systems, but the outcome was probably influenced by the specific training stimulus and the individual differences [2,83].

It has been documented that the LEAs’ training programs, even though they place so much emphasis on physical training by devoting numerous hours during the years in the academy, sometimes these programs, due to the one-size fits-all approach, may lack a scientific base of formal progression that probably hinders cadets’ development [18,19,84]. In order to cover the overall class’ fitness levels, most often intensities are set based on the less fit cadets, resulting in under-training for the fitter ones [85]. Less often, the intensities are prescribed based on the more fit cadets, as in this case over-training and over-use injuries are more likely to happen to the vast majority of the cadets [85,86,87]. For example, it has been shown that during a standardized run, cadets’ heart rate responses can vary greatly within the running group, pointing out that different training stimuli have been applied to cadets within this standardized exercise stimulus [51,52]. A suggestion to address such a disparity between the academic years is to introduce physical and wellness education curricula to develop health habits based on individualized exercise prescription programs, something that this particular LEA employs for the last year in the academy. Alternatively, an ability-based training program has been found to be equivalent or even superior to the one-size-fits- all approach, presenting less injury risks for the cadets [18] and a time-efficient way compared to traditional models in respect to aerobic conditioning [18]. Therefore, since the results of this study are mostly in favor of individualized exercise prescription, due to the observed differences between classes and the great variability in the examined variables, it may be more beneficial for cadets’ wellbeing during the academy and later within their careers as police officers to have this educational approach introduced earlier in the academy [6,15,16,34].

Regarding sex comparisons, males were heavier and taller than the female cadets. Males significantly increased their weight throughout the years in the academy (freshman vs. sophomore, freshman vs. junior, freshman vs. senior), while the females remained stable for the first two years, then became heavier in the third year and they were their lightest in the last fourth year. These body weight changes may have a practical implication considering the physical demands of police work and a successful outcome [88], but since no body composition assessment was performed, it is difficult to attribute these changes solely or partially in the applied training program or any other related lifestyle factors during the academy years.

Regarding sex fitness performance comparisons, on average, male cadets outperformed the female ones in every physical testing. This sex difference is aligned with previous research [23,24,89,90], especially when we consider that males tend to be more muscular and in absolute values, present higher physiological capacities than the females [23,89,91,92,93,94]. A study on custody assistants from a law enforcement agency showed that males scored higher than females at handgrip, push-ups, sit-ups and were faster with a higher maximum oxygen consumption, as documented with the 1.5-mile run [23]. On the other hand, these sex differences highlight the importance of having fitness standards normalized for sex [95] and sex-specific training adjustments [7]. It is possible that the one-size-fits-all approach imposes greater physiological (e.g., load and intensity) and psychological stress on female cadets than male ones [25,96,97], or could lead to increased injury rates [98], an issue that can be addressed when an individualized or ability-based exercise program is applied [7]. Based on the previous literature and supported by the findings of this study, females should probably focus on improving their physical fitness even prior to their induction to the academy and chance to support normalizing fitness standards for sex/gender [21,23].

Due to the nature of this retrospective analysis, this study is not free of limitations. This study incorporated one LEA from Serbia that may operate under different laws and regulations, even within the same country [40]. It was assumed that the size of the classes and fitness levels of cadets were representative only for this LEA [21]. It is out of the researchers’ knowledge whether or not the results of the fitness assessments were used to design and amend the training program, either the one-size-fits-all or the individualized one, a practice that is very common in sports and strength and conditioning [99]. The unequal size of the classes may have influenced the outcomes, but this could not have been controlled as no report on why the classes were larger or smaller was provided (e.g., separation) [6]. The unequal class size, especially the sophomores (n = 91), would have been a prohibited factor for employing an individualized exercise program as opposed to the senior year class that had only 15 cadets. Another potential limiting factor that must be noted is the availability and space for training between the years [35], something that was assumed to be the same across the years. The training hours devoted and or the workday schedule per year of this LEA was something out of the researchers’ control. It may be possible that the training hours did not meet the minimum standards or there was a discrepancy between the training hours per academic year due to the curriculum. We are not in a position to know this LEAs’ specific training mandates, but we assumed that all the training programming was based on sound exercise related scientific principles, since it was performed by certified exercise specialists [17,80,83]. Moreover, no attempt was made to compare the fitness test scores of this study with other LEAs’ fitness scores worldwide, as this was not the aim of this study.

Besides all of these limitations, this study captured specific trends that are happening in a specific LEA and provided information that can be used not only to change this LEAs’ curriculum and training practices, but also to support the advocates of a change in LEAs’ current training models even more. This information can be used to develop a curriculum that prepare LEOs to successfully complete their academy training, but also embrace a lifestyle that promotes health and wellness that can be maintained even upon their graduation and during their whole professional career. Even though there is a disparity between the sexes/genders in LEA research [8,10,21,23,24,40], this study had almost equal sex sizes adding in the body of literature and giving great value to practitioners in this field [10,100,101]. Future randomized studies need to analyze the fitness levels following cadets within the academy and evaluate the effectiveness of the individualized and ability-based training models in comparison to the traditional military style of one-size-fits-all approach.

## 6. Conclusions

In general, this study presented differences in the fitness scores of police cadets throughout their four years of training, suggesting that the one-size-fits-all approach as employed throughout the years one to three, may be less than optimal [16]. The self-individualized exercise prescription of the fourth/senior year elicited greater fitness-related scores. Therefore, this may indicate the need for individualized and/or ability-based training [18]. It is important for LEAs and exercise staff to recognize both the potential fitness and the inherent sex/gender differences among cadets. Doing as such, the individualized and ability-based training program is probably a better approach to meet both the LEAs’ and cadets’ fitness related objectives [30].

## Figures and Tables

**Table 1:** Descriptive Statistics for Age, Height and Weight per Academic Years and Sex.

Academic Standing (Years)	Sex		*N*	Minimum	Maximum	Mean	Std. Deviation
Freshman	Male	Age (years)	15	19.0	21.0	19.5	0.8
		Height (cm)	15	174.0	201.0	181.7	7.9
		Weight (kg)	15	65.0	100.0	78.5	10.4
	Female	Age (years)	15	19.0	20.0	19.3	0.5
		Height (cm)	15	164.5	182.0	169.8	4.8
		Weight (kg)	15	55.0	80.0	63.5	6.5
Sophomore	Male	Age (years)	54	20.0	24.0	20.4	0.8
		Height (cm)	54	173.0	198.0	183.1	6.1
		Weight (kg)	54	67.0	100.0	81.5	8.1
	Female	Age (years)	37	20.0	24.0	20.8	0.9
		Height (cm)	37	165.0	180.0	170.1	3.8
		Weight (kg)	37	54.0	75.0	63.6	6.0
Junior	Male	Age (years)	16	21.0	25.0	21.6	1.0
		Height (cm)	16	175.0	196.0	183.1	6.2
		Weight (kg)	16	73.0	108.0	86.0	9.1
	Female	Age (years)	22	21.0	27.0	22.0	1.3
		Height (cm)	22	165.0	188.0	172.0	5.8
		Weight (kg)	22	53.0	85.0	65.2	7.4
Senior	Male	Age (years)	10	22.0	24.0	22.8	0.8
		Height (cm)	10	171.0	194.0	186.3	6.7
		Weight (kg)	10	77.0	102.0	89.2	7.9
	Female	Age (years)	5	22.0	23.0	22.4	0.5
		Height (cm)	5	166.0	174.0	169.6	3.0
		Weight (kg)	5	57.0	69.0	62.8	5.4

**Table 2:** Descriptive Statistics for examined variables.

	Academic Standing (Years)	Sex	Mean	Std. Deviation	*N*
Absolute Bilateral Handgrip Strength (kg)	Freshman	Male	125.78	13.45	15
		Female	77.96	8.83	15
		Total	101.87	26.77	30
	Sophomore	Male	120.55	12.44	54
		Female	74.23	8.80	37
		Total	101.71	25.41	91
	Junior	Male	123.98	9.70	16
		Female	76.84	6.36	22
		Total	96.68	24.85	38
	Senior	Male	125.66	6.51	10
		Female	77.56	9.52	5
		Total	109.63	24.58	15
	Total	Male	122.49	11.77	95
		Female	75.87	8.24	79
		Total	101.32	25.45	174
Deadlift max (kg)	Freshman	Male	167.97	22.74	15
		Female	96.43	19.14	15
		Total	132.20	41.84	30
	Sophomore	Male	170.10	20.36	54
		Female	102.74	15.81	37
		Total	142.71	38.09	91
	Junior	Male	176.74	13.75	16
		Female	109.31	10.57	22
		Total	137.70	35.75	38
	Senior	Male	177.77	14.56	10
		Female	118.86	25.26	5
		Total	158.13	33.84	15
	Total	Male	171.69	19.32	95
		Female	104.39	16.66	79
		Total	141.14	38.17	174
Half-squat max (kg)	Freshman	Male	165.75	21.04	15
		Female	93.29	16.10	15
		Total	129.52	41.20	30
	Sophomore	Male	181.43	21.86	54
		Female	107.23	15.55	37
		Total	151.26	41.49	91
	Junior	Male	184.41	19.38	16
		Female	121.48	10.92	22
		Total	147.98	34.80	38
	Senior	Male	185.22	16.45	10
		Female	114.64	14.36	5
		Total	161.69	37.67	15
	Total	Male	179.86	21.47	95
		Female	109.02	17.20	79
		Total	147.70	40.43	174
Standing long jump (cm)	Freshman	Male	242.47	19.03	15
		Female	192.07	13.92	15
		Total	217.27	30.42	30
	Sophomore	Male	229.69	12.94	54
		Female	177.38	13.37	37
		Total	208.42	28.94	91
	Junior	Male	234.44	11.45	16
		Female	183.50	15.18	22
		Total	204.95	28.87	38
	Senior	Male	241.90	15.13	10
		Female	180.60	9.15	5
		Total	221.47	32.64	15
	Total	Male	233.79	14.83	95
		Female	182.08	14.63	79
		Total	210.31	29.71	174
Push-ups in total (#)	Freshman	Male	12.28	1.63	15
		Female	6.80	2.91	15
		Total	9.54	3.62	30
	Sophomore	Male	12.50	1.31	54
		Female	6.70	2.07	37
		Total	10.14	3.30	91
	Junior	Male	12.09	1.05	16
		Female	7.95	1.40	22
		Total	9.70	2.42	38
	Senior	Male	12.86	1.05	10
		Female	7.20	.84	5
		Total	10.97	2.92	15
	Total	Male	12.44	1.30	95
		Female	7.10	2.09	79
		Total	10.01	3.16	174
Sit-ups in 30 sec (#)	Freshman	Male	27.67	3.31	15
		Female	23.07	2.74	15
		Total	25.37	3.79	30
	Sophomore	Male	25.20	2.48	54
		Female	22.59	1.77	37
		Total	24.14	2.56	91
	Junior	Male	27.75	2.05	16
		Female	23.55	2.04	22
		Total	25.32	2.91	38
	Senior	Male	26.60	1.84	10
		Female	23.20	1.64	5
		Total	25.47	2.39	15
	Total	Male	26.17	2.73	95
		Female	22.99	2.05	79
		Total	24.72	2.91	174
Pull-ups in total (#)	Freshman	Male	22.61	10.08	15
		Female	1.20	1.32	15
		Total	11.91	12.98	30
	Sophomore	Male	18.10	5.84	54
		Female	1.46	1.92	37
		Total	11.33	9.44	91
	Junior	Male	16.16	4.37	16
		Female	2.73	2.00	22
		Total	8.38	7.43	38
	Senior	Male	16.47	2.64	10
		Female	1.80	0.45	5
		Total	11.58	7.47	15
	Total	Male	18.31	6.50	95
		Female	1.78	1.87	79
		Total	10.81	9.62	174
12-min Cooper test (m)	Freshman	Male	2751.00	151.80	15
		Female	2268.00	158.53	15
		Total	2509.50	289.12	30
	Sophomore	Male	2694.07	193.04	54
		Female	2117.70	219.04	37
		Total	2459.73	349.54	91
	Junior	Male	2797.19	136.63	16
		Female	2175.45	141.18	22
		Total	2437.24	340.08	38
	Senior	Male	2811.00	85.04	10
		Female	2215.00	204.63	5
		Total	2612.33	318.10	15
	Total	Male	2732.74	174.31	95
		Female	2168.48	193.52	79
		Total	2476.55	335.82	174

# Denotes number of performed repetitions for the respective exercises.

**Table 3:** Multiple Comparisons for Academic Years.

Dependent Variable		(I) Academic Standing (Years)	(J) Academic Standing (Years)	Mean Difference (I−J)	Std. Error	Sig.	95% Confidence Interval	
							Lower Bound	Upper Bound
Deadlift max (kg)	Bonferroni	Freshman	Sophomore	−10.51 *	3.76	0.03	−20.56	−0.47
			Junior	−5.50	4.36	1.00	−17.15	6.15
			Senior	−25.93 *	5.65	0.00	−41.02	−10.85
		Sophomore	Freshman	10.51 *	3.76	0.03	0.47	20.56
			Junior	5.01	3.45	0.89	−4.20	14.23
			Senior	−15.42 *	4.98	0.01	−28.71	−2.13
		Junior	Freshman	5.50	4.36	1.00	−6.15	17.15
			Sophomore	−5.01	3.45	0.89	−14.23	4.20
			Senior	−20.43 *	5.45	0.00	−34.98	−5.89
		Senior	Freshman	25.93 *	5.65	0.00	10.85	41.02
			Sophomore	15.42 *	4.98	0.01	2.13	28.71
			Junior	20.43 *	5.45	0.00	5.89	34.98
Half-squat max (kg)	Bonferroni	Freshman	Sophomore	−21.74 *	3.84	0.00	−32.00	−11.49
			Junior	−18.46 *	4.46	0.00	−30.36	−6.56
			Senior	−32.17 *	5.77	0.00	−47.58	−16.77
		Sophomore	Freshman	21.74 *	3.84	0.00	11.49	32.00
			Junior	3.29	3.52	1.00	−6.12	12.70
			Senior	−10.43	5.08	0.25	−24.01	3.15
		Junior	Freshman	18.46 *	4.46	0.00	6.56	30.36
			Sophomore	−3.29	3.52	1.00	−12.70	6.12
			Senior	−13.72	5.56	0.09	−28.57	1.14
		Senior	Freshman	32.17 *	5.77	0.00	16.77	47.58
			Sophomore	10.43	5.08	0.25	−3.15	24.01
			Junior	13.72	5.56	0.09	−1.14	28.57
Standing long jump (cm)	Bonferroni	Freshman	Sophomore	8.85 *	2.94	0.02	1.01	16.69
			Junior	12.32 *	3.41	0.00	3.22	21.41
			Senior	−4.20	4.41	1.00	−15.98	7.58
		Sophomore	Freshman	−8.85 *	2.94	0.02	−16.69	−1.01
			Junior	3.47	2.69	1.00	−3.72	10.66
			Senior	−13.05 *	3.89	0.01	−23.43	−2.67
		Junior	Freshman	−12.32 *	3.41	0.00	−21.41	−3.22
			Sophomore	−3.47	2.69	1.00	−10.66	3.72
			Senior	−16.52 *	4.25	0.00	−27.88	−5.16
		Senior	Freshman	4.20	4.41	1.00	−7.58	15.98
			Sophomore	13.05 *	3.89	0.01	2.67	23.43
			Junior	16.52 *	4.25	0.00	5.16	27.88
Sit-ups in 30 s (#)	Bonferroni	Freshman	Sophomore	1.22	0.49	0.08	−0.08	2.53
			Junior	0.05	0.57	1.00	−1.46	1.56
			Senior	−0.10	0.73	1.00	−2.05	1.85
		Sophomore	Freshman	−1.22	0.49	0.08	−2.53	0.08
			Junior	−1.17 *	0.45	0.06	−2.37	0.02
			Senior	−1.32	0.65	0.25	−3.05	0.40
		Junior	Freshman	−0.05	0.57	1.00	−1.56	1.46
			Sophomore	1.17 *	0.45	0.06	−0.02	2.37
			Senior	−0.15	0.71	1.00	−2.04	1.73
		Senior	Freshman	0.10	0.73	1.00	−1.85	2.05
			Sophomore	1.32	0.65	0.25	−0.40	3.05
			Junior	0.15	0.71	1.00	−1.73	2.04
12-min Cooper test (m)	Bonferroni	Freshman	Sophomore	49.77	37.65	1.00	−50.75	150.30
			Junior	72.26	43.67	0.60	−44.35	188.88
			Senior	−102.83	56.55	0.42	−253.83	48.16
		Sophomore	Freshman	−49.77	37.65	1.00	−150.30	50.75
			Junior	22.49	34.54	1.00	−69.74	114.71
			Senior	−152.61 *	49.83	0.02	−285.67	−19.55
		Junior	Freshman	−72.26	43.67	0.60	−188.88	44.35
			Sophomore	−22.49	34.54	1.00	−114.71	69.74
			Senior	−175.10 *	54.53	0.01	−320.70	−29.50
		Senior	Freshman	102.83	56.55	0.42	−48.16	253.83
			Sophomore	152.61 *	49.83	0.02	19.55	285.67
			Junior	175.10 *	54.53	0.01	29.50	320.70

Based on observed means. The error term is Mean Square (Error) = 31,975.41. * The mean difference is significant at the 0.05 level. # Denotes number of performed repetitions for the respective exercises.

**Table 4:** Sex Pairwise Comparisons.

Dependent Variable	(I) Sex	(J) Sex	Mean Difference (I−J)	Std. Error	Sig.	95% Confidence Interval for Difference ^b^	
						Lower Bound	Upper Bound
Absolute Bilateral Handgrip Strength (kg)	Male	Female	47.35 *	1.97	<0.0001	43.45	51.24
	Female	Male	−47.35 *	1.97	<0.0001	−51.24	−43.45
Deadlift max (kg)	Male	Female	66.31 *	3.42	<0.0001	59.56	73.07
	Female	Male	−66.31 *	3.42	<0.0001	−73.07	−59.56
Half-squat max (kg)	Male	Female	70.04 *	3.49	<0.0001	63.14	76.94
	Female	Male	−70.04 *	3.49	<0.0001	−76.94	−63.14
Standing long jump (cm)	Male	Female	53.74 *	2.67	<0.0001	48.46	59.01
	Female	Male	−53.74 *	2.67	<0.0001	−59.01	−48.46
Push-ups in total (#)	Male	Female	5.27 *	0.32	<0.0001	4.63	5.90
	Female	Male	−5.27 *	0.32	<0.0001	−5.90	−4.63
Sit-ups in 30 s (#)	Male	Female	3.70 *	0.44	<0.0001	2.83	4.58
	Female	Male	−3.70 *	0.44	<0.0001	−4.58	−2.83
Pull-ups in total (#)	Male	Female	16.54 *	0.92	<0.0001	14.72	18.35
	Female	Male	−16.54 *	0.92	<0.0001	−18.35	−14.72
12-min Cooper test (m)	Male	Female	569.28 *	34.25	<0.0001	501.66	636.89
	Female	Male	−569.28 *	34.25	<0.0001	−636.89	−501.66

Based on marginal means. * The mean difference is significant at the 0.05 level. ^b^ Adjustment for multiple comparisons: Bonferroni. # Denotes number of performed repetitions for the respective exercises.

## Data Availability

Data are available upon request.
